# Author Correction: Fossilized solidification fronts in the Bushveld Complex argue for liquid-dominated magmatic systems

**DOI:** 10.1038/s41467-020-17671-x

**Published:** 2020-07-24

**Authors:** Willem Kruger, Rais Latypov

**Affiliations:** School of Geosciences, University of the Witwatersrand, Johannesburg, South Africa

**Keywords:** Geochemistry, Economic geology, Mineralogy, Petrology

Correction to: *Nature Communications* 10.1038/s41467-020-16723-6; published online 9 June 2020.

The original version of this Article contained an error in the title, which was previously incorrectly given as ‘Fossilized solidifications fronts in the Bushveld Complex argues for liquid-dominated magmatic systems’. The correct version states ‘Fossilized solidification fronts in the Bushveld Complex argue for liquid-dominated magmatic systems’.

This has been corrected in both the PDF and HTML versions of the Article.

