# Supplementary material for: Fossilized solidifications fronts in the Bushveld Complex argues for liquid-dominated magmatic systems
Source: Nat Commun. 2020 Jun 9;11:2909. doi: 10.1038/s41467-020-16723-6 (PMC7283281; doi:10.1038/s41467-020-16723-6)
Supplement: Supplementary file 1 — Supplementary Information [file 41467_2020_16723_MOESM1_ESM.pdf]

Supplementary Information Files for paper entitled “Discovery of fossilized solidification fronts in the Bushveld Complex argues for liquid-dominated magmatic systems”

Kruger and Latypov (2020)

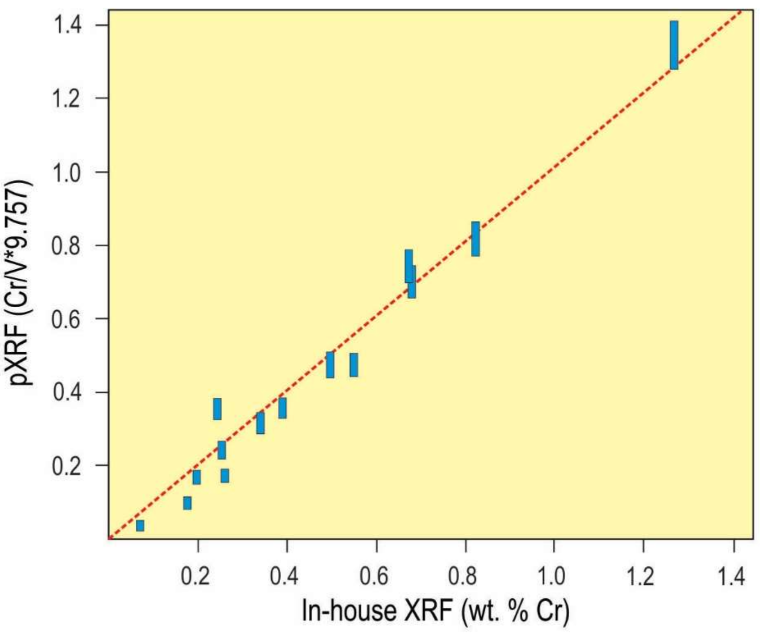

Supplementary information Fig. 1. Calibration curve for the portable X-ray fluorescence spectrometer. Hand samples collected from the MML were analysed via the pXRF except for the sample with the highest Cr concentration which was pulverised prior to analysis (high Cr concentration gradients are present in samples with very high Cr concentrations and the sample needs to be homogenized before any chemical analysis can be performed). The vertical spacing of the blue bars represents the 2σ analytical uncertainty of the pXRF analysis. In-house XRF data[60] were obtained from pure magnetite separates. Because the pXRF data does not come from pure magnetite separates, some difference between the pXRF and XRF data is to be expected.

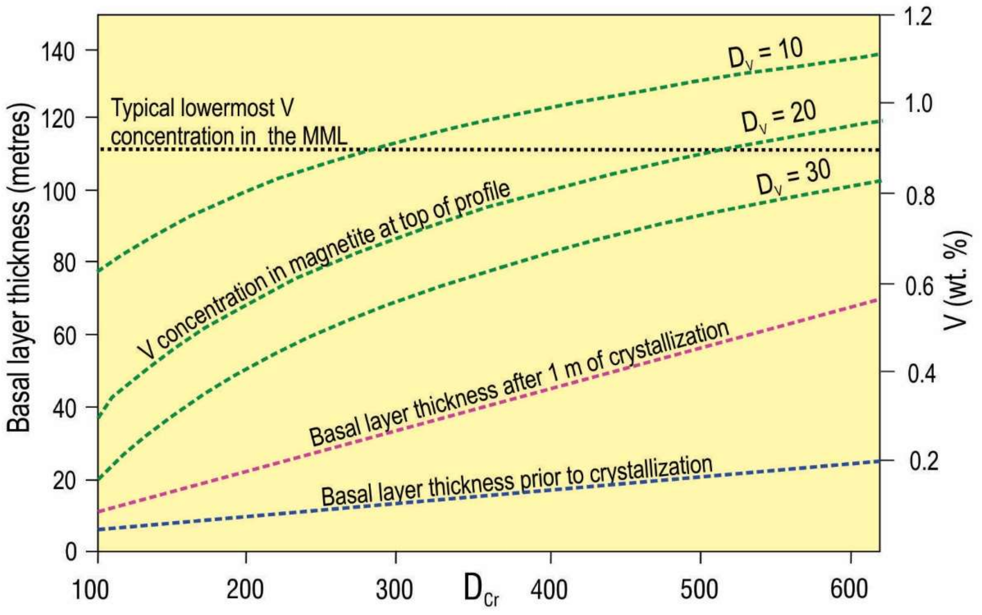

Supplementary Information Fig. 2. Constraints on the basal melt layer thickness depending on the selected D value for Cr. The diagram should be read in the following way: first select a partition coefficient for Cr in magnetitite on the x-axis. Trace a line directly upwards until the blue line is intercepted. From this point the initial basal layer thickness before crystallization can be read off on the y-axis on the left. Trace the line further upwards until in intercepts the purple line. This allows the final thickness of the basal melt layer to be read off the y-axis after the deposition of 1 metre of magnetitite assuming the basal melt layer grows incrementally due to continued magma addition. Continue tracing the line upwards to intercept one of the green curves depending on the preferred D value for V (10, 20, or 30). This allows the determination of the final V concentration of the MML based on a starting V concentration that can be read off on the y-axis on the right (Methods). By using the entire range of experimentally determined D values for Cr in magnetite in basaltic melt[28], the initial basal layer thickness is unlikely to be more than 6 metres and less than 27 metres thick to accurately describe the distribution of Cr in the MML. After 1 m of magnetitite crystallization, the basal layer has grown to a thickness of between 12 and 71 metres thick due to the addition of melt to the basal layer. The upper curves (green) indicate V concentration at the upper part of the MML assuming a starting V concentration in magnetite of 1.23 wt. % compared to the typical lowermost V concentration recorded in this layer[60] (the typical lowermost V concentration is indicated by the black horizontal stippled line). To prevent the V concentration from dropping below the typical lowermost value, and assuming a D for V in magnetite of about 20, a D value of 525 is needed for Cr in magnetite. This requires an initial basal melt layer thickness to be of about 20 metres. These are the final modelling parameters used (Fig. 6c and 7b) (Methods).
