# Supplementary material for: Fossilized solidifications fronts in the Bushveld Complex argues for liquid-dominated magmatic systems
Source: Nat Commun. 2020 Jun 9;11:2909. doi: 10.1038/s41467-020-16723-6 (PMC7283281; doi:10.1038/s41467-020-16723-6)
Supplement: Supplementary file 3 — Description of Additional Supplementary Files [file 41467_2020_16723_MOESM3_ESM.pdf]

Description of Additional Supplementary Files

File Name: Supplementary Data 1
Description: **Geochemical data.** Geochemical data for the studied vertical profiles across a magnetite layer

File Name: Supplementary Data 2
Description: **Geochemical modelling.** Geochemical modelling of Cr and V distribution in a magnetite layer

File Name: Supplementary Data 3
Description: **Boundary layer properties.** Properties of a compositional boundary layer at magnetite-liquid interface

File Name: Supplementary Data 4
Description: **MELTS run.** MELTS run for a magnetite-only-saturated melt parental to a magnetite layer

File Name: Supplementary Movie 1
Description: **Animation of magnetite growth based on the distribution of Cr in the layer.** Magnetite starts nucleating and growing in three concentrated spots along the floor of the chamber in structures called “growth nodes”. Growth proceeds laterally until nodes coalesce into a planar solidification front. The animation is based on the results obtained in the profile shown in Figure 3a and b.
